# Supplementary material for: AnnoView enables large-scale analysis, comparison, and visualization of microbial gene neighborhoods
Source: Brief Bioinform. 2024 May 14;25(3):bbae229. doi: 10.1093/bib/bbae229 (PMC11094555; doi:10.1093/bib/bbae229)
Supplement: SuppFigures_BIB_bbae229 [file suppfigures_bib_bbae229.pdf]

SUPPLEMENTARY FIGURES

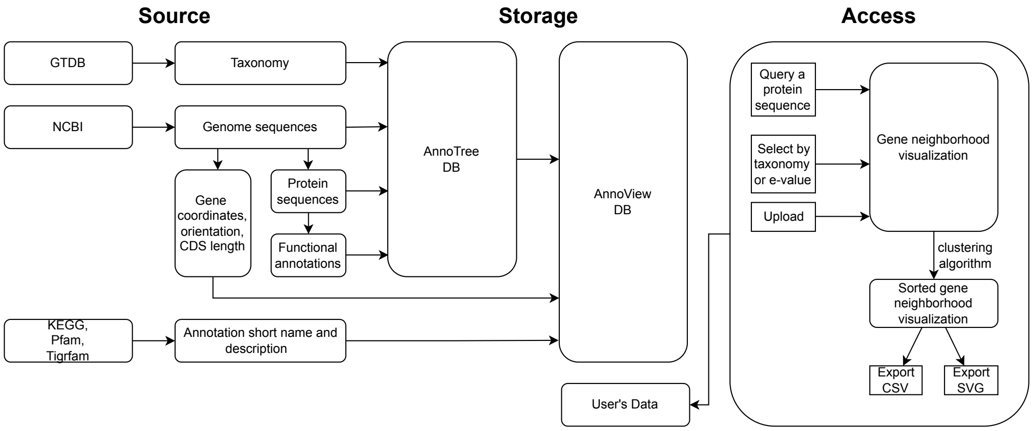

Supplementary Figure 1. Software architecture diagram showing the components of AnnoView.

Algorithm 1: Smith-Waterman similarity calculation between two genomes

SimCal $(g1, g2)$;
Input : Genome g1, Genome g2
Output: Weighted similarity between g1 and g2
Scoring matrix $H \leftarrow$ Smith-Waterman(g1,g2)
Sim $\leftarrow H_{length(g1),lengh(g2)}$
K $\leftarrow$ index(max(H))
return Sim/K

Algorithm 2: Calculate similarities among all genomes

Similarity (G);
Input : Genomes G
Output: Similarity score matrix H
For every pair $g1, g2 \in G$
i $\leftarrow$ index of center gene in g1
j $\leftarrow$ index of center gene in g2
Leftg1 $\leftarrow$ g1[0:i]
Leftg2 $\leftarrow$ g2[0:j]
Rightg1 $\leftarrow$ g1[i+1:]
Rightg2 $\leftarrow$ g2[i+j:]
Sim $\leftarrow$ SimCal(Leftg1,leftg2)+ SimCal(Rightg1,Rightg2)
return H

Algorithm 3: Reorder genomes for cluster visualization based on a target center gene.

Center Sort (G);
Input : Genomes G, a list of genomes, each genome is a list of genes
Output: Order O
Similarity score matrix H $\leftarrow$ Similarity(G)
H $\leftarrow$ Min-Max Normalization(H): $1 - \frac{H_{ij} - minH}{max(H) - min(H)}$
H $\leftarrow$ hierarchicalClustring(H)
O $\leftarrow$ FastOptimalLeafOrdering(H)
return O

Supplementary Figure 2. Pseudocode for the clustering algorithm that sorts gene neighborhoods based on a target center gene.

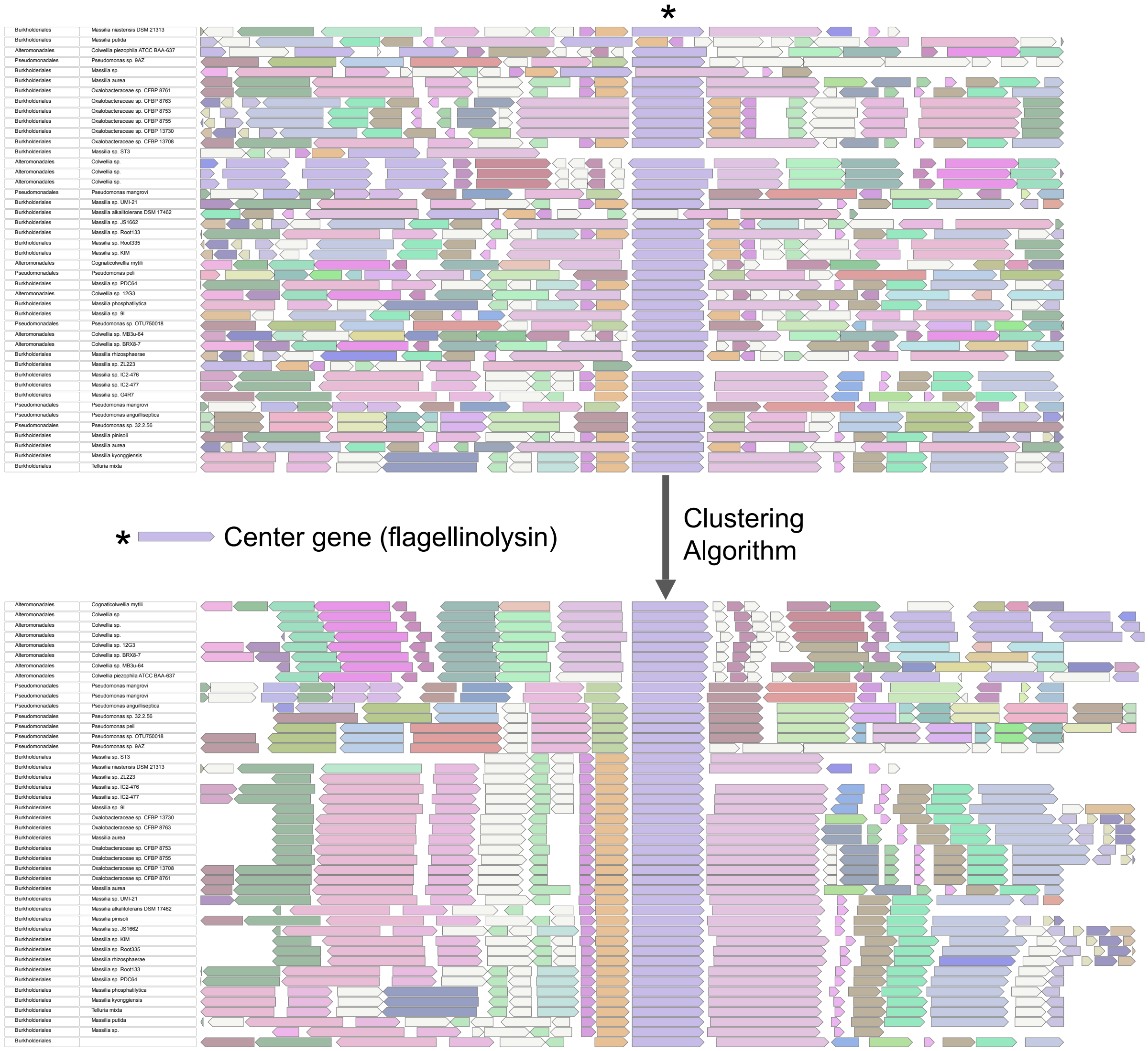

Supplementary Figure 3. An example demonstrating the gene neighborhood clustering algorithm. A pre-clustered set of gene neighborhoods is shown above. The neighborhoods are then clustered and sorted (below) based on flagellinolysin [69] selected as the center gene (indicated by asterisk). Gene neighborhoods are clustered based on gene composition, which is defined based on their lists of functional annotations. After sorting, patterns emerge which illustrate evolutionary groupings of genomic regions based on their common ancestry.
