# Supplementary material for: AnnoView enables large-scale analysis, comparison, and visualization of microbial gene neighborhoods
Source: Brief Bioinform. 2024 May 14;25(3):bbae229. doi: 10.1093/bib/bbae229 (PMC11094555; doi:10.1093/bib/bbae229)
Supplement: SupTable1_bbae229 [file suptable1_bbae229.docx]

**Supplementary Table 1.** Comparison of gene neighborhood analysis/visualization tools.

| **Tool** | **Webserver available?** | **Supported genomes/taxa** | **Input** | **Max # tracks for display** | **Gene annotation method** | **Sorting/ranking method** |
| --- | --- | --- | --- | --- | --- | --- |
| AnnoView | Yes | All genomes in GTDB (bacteria and archaea; Search GTDB mode) or any genomic region (Upload dataset mode) | GBK files, GFF files, CSV files, or protein sequences | 500 | KEGG, Pfam, TIGRFAM | Based on Smith-Waterman and hierarchical clustering |
| CAGECAT | Yes | All nt/nr sequences in the NCBI (sequence hits then queried against Identical Protein Groups – Remote search mode) or fungi and prokaryotes (HMM search mode) | Nucleotide/protein sequences, GBK files, NCBI accessions, or HMM profile identifiers | 50 | Defines gene groups based on BLAST similarity | Based on the number of matching proteins to the query cluster |
| EvolClustDB | Yes | 838 eukaryotic species including fungi, plants, metazoans, insects, and protists | NCBI/UniProt/TAIR gene ID, species name, gene cluster name (EvolclustDB cluster ID), or protein sequences | 150 | Defines gene groups using all-against-all BLASTP search | Based on species trees obtained from NCBI (subsequently pruned) for all but fungi which was generated in a previous analysis |
| fast.genomics | Yes | 6,377 bacterial and archaeal genomes (1 per genus – main database) and up to 10 genomes per prokaryotic species (order-level database) | Locus tags, UniProt ID or name, PDB entry, protein sequences, or a taxon | 200 | Defines gene groups using LAST | Ranked by descending order of bit score or gene phylogenetic tree (generated with MUSCLE 3 and FastTree 2) |
| FlaGs and WebFlaGs | Yes (WebFlaGs) | All Refseq sequences, a reduced database with 13548 bacterial, 467 archaeal and 10449 viral genomes, or (local install only) any Refseq formatted genomes | Protein sequences or NCBI Refseq protein accessions | 200 (WebFlaGs) | Detects homologous proteins using Jackhmmer | Based on user-inputted order or protein phylogenetic tree (generated with ETE 3) |
| Gbrowse (no longer supported) | No | Any GFF or BED formatted sequences | GFF, BED, and/or SAM/BAM files | No set maximum | From GFF or BED files | - |
| gcluster | No | Any GenBank formatted genome | GBK files and a list of locus tags from a gene of interest in each genome | No set maximum | Detects homologs using OrthoMCL; also accepts homology detection results from user | Based on user-inputted order or based on optional phylogenetic tree provided by user |
| gcsnap | No | Any NCBI sequence (inputs mapped to protein EntrezID) | At least two target sequence identifiers or a cluster file (CLANS format) from UniprotKB, UniRef, Ensemble, or Entrez | No set maximum | Defines protein families using all-against-all BLASTP or MMseqs2 searches and annotates with Phobius, TMHMM, and features found in UniprotKB, SWISS-MODEL | Based on user-inputted order, taxonomic clustering from NCBI, by a user-provided phylogenetic tree, or (Advanced mode) PaCMAP |
| GeCoViz | Yes | 12,221 prokaryotic genomes | Gene names, protein sequences, orthologous group IDs from eggNOG v5, KEGG or Pfam IDs | 400 | COG, KEGG, Pfam, and orthology annotations from eggNOG v5 | Based on species tree obtained from NCBI |
| genegraphics | Yes | Any GenBank or TSV formatted sequence | NCBI gene/protein ID, UniprotKB accession, gene symbol paired with a NCBI genome ID, genomic region range, GBK file, SEED TSV dataset or GizmoGene TSV dataset | No set maximum | NCBI annotations | - |
| Genespy | No | Not restricted | NCBI identifiers, BLASTP output files, GBK files, or direct searches of local GFF files | 330 | Annotations from local GFF files | Based on user-inputted order or based on optional phylogenetic tree provided by user |
| Genome Context Viewer | Yes (currently only for Legume Information System, Legume Federation Outgroups, Medicago, Phaseolus, Cicer, and Solunum Genomes) | Not restricted | Gene names or chromosome regions | No set maximum | Function annotation from custom database | Smith-Waterman and Repeat local alignment algorithms to cluster gene neighborhoods |
| Microbesonline | Yes | 3707 genomes including bacteria, archaea and fungi | A list of genomes and either nucleotide/protein sequences or a gene name/keyword | 400 | Detects homologs based on all-against-all FastBLAST or BLAST (slow mode) and  gene family, domain architecture, EC number, GO terms, TIGRFAM, Superfamily, SMART, Pfam, Panther, PirSF, Gene3D, COG, and PDB annotations are also provided. Characterized genes (via UniProt and RegTransBase) are also indicated | Based on gene tree created using FastTree |
| syntax |  | Prokaryotes from NCBI | A list of genomes and either protein sequences | 120 | Detect homologs by the Smith-Waterman-Gotoh algorithm | Rank by normalized sequence similarity scores |
| SynView (not currently available) | No | Not restricted | GFF or BED files | No set maximum | Detects homologs based on OrthoMCL clustering and provides annotations from GFF or BED files | - |
| TREND | Yes | 125,000+ prokaryotic genomes | Protein sequence, multiple sequence alignment, NCBI RefSeq IDs, locus tags, MiST IDs, or a phylogenetic tree (Newick format) | No set maximum | Pfam, CDD, TMHMM, SEG, | Based on protein phylogenetic tree generated with MAFFT, then FastTree or MEGA |
